# Electrophysiologically informed spiking neural networks for fish-inspired navigation with boundary vector cells and hydrostatic pressure cues

**DOI:** 10.1016/j.isci.2026.115824

**Published:** 2026-04-20

**Authors:** Lear Cohen, Hadar Cohen Duwek, Elishai Ezra Tsur

**Affiliations:** 1 The Neuro-biomorphic Engineering Lab (NBEL), The Open University of Israel, Raanana, Israel

**Keywords:** behavioral neuroscience, systems neuroscience, cognitive neuroscience

## Abstract

Spatial memory is a fundamental cognitive capacity governing the mental encoding of spatial information, which can be used to navigate through intricate terrains. While most navigation models rely on place- and grid cells as key building blocks, in fish, the largest vertebrate class, the neural basis of navigation was suggested to primarily comprise boundary vector cells (BVCs) and hydrostatic pressure (HP) cues. In this work, we used experimental neural data recordings from the telencephalon of the goldfish to implement a neuromorphic (brain-inspired) spiking neural network-based navigation framework. BVCs were used for collision avoidance and HP for trajectory control toward the target. We show that the model supports reliable goal-directed navigation in a depth-constrained task without requiring explicit place-cell position encoding. Navigation performance emerges from the interaction between boundary-related signals, HP cues, and a fixed initial directional bias. These results provide a computational account of how biologically grounded cues can support efficient navigation under constrained sensory assumptions.

## Highlights

• Biologically grounded spiking neural design calibrated against electrophysiological data
• Boundary-vector-cell navigation without place or grid cells
• Hydrostatic pressure enables depth-guided spatial search
• Comparable performance to classical planners without map representation

## Graphical abstract

**Figure:**
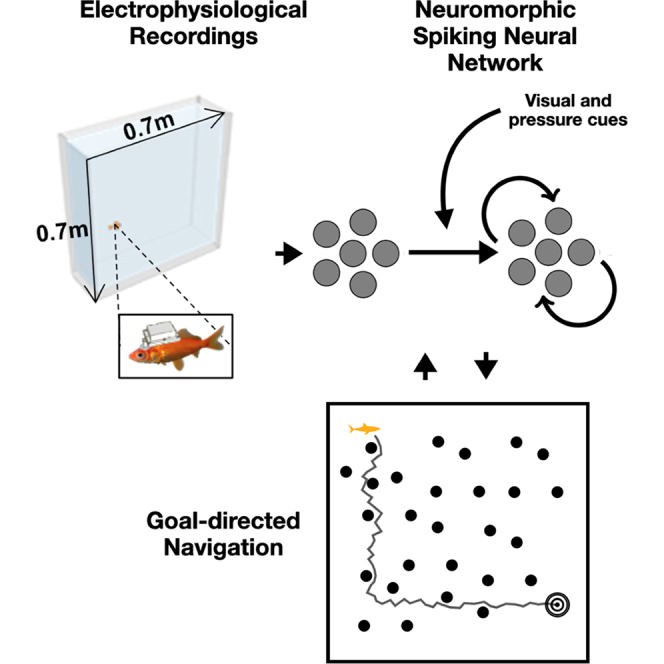

## Introduction

Almost all animal species use spatial memory to allocate food and shelter, or to avoid predators. This cognitive ability includes neuronal representation of environmental spatial features, such as the position of self, others, and salient features of the environment. The neuronal building blocks of the navigation system were investigated in many animal species.^1^^,^^2^^,^^3^^,^^4^^,^^5^ Two standard key building blocks, which were found in various species, are place^6^ and grid cells.^7^ While place cells enable the derivation of one’s current position, grid cells provide a measure of traveled distances. Together, these building blocks are fundamental in many models of neural navigation systems.^8^^,^^9^^,^^10^

However, studies that aimed to better resemble the natural environment of the studied animals implied a different picture. For instance, the typical Gaussian place cells shown in the controlled environments used in closed laboratory environments have changed their spatial tuning in large realistic environments.^11^ Moreover, the hexagonal symmetry of the 2D grid cell pattern differed from what was expected when recorded in a 3D setup.^12^ Finally, it was suggested that more complex models are needed to successfully navigate a natural environment.^10^^,^^13^

Fish are the largest and most diverse group of vertebrates. They were shown to successfully navigate in laboratory settings using both allocentric and egocentric coordinate systems,^14^^,^^15^ as well as in natural environments, repeating their trajectories over days to reach their feeding sites.^16^ Further, electrophysiological studies in the goldfish telencephalon have not found typical place or grid cells resembling the mammalian hippocampal neurons: Instead, it was suggested that the building blocks of the goldfish navigation system are boundary vector cells (BVCs^17^; encoding distance and direction from salient environmental features. These units’ firing rate gradually decreases in accordance with the distance from salient features found in a specific direction. Additional behavioral^18^^,^^19^ and electrophysiological^17^ studies have also suggested that fish use hydrostatic pressure (HP) to encode vertical positions to find food. Together with the kinematical cell types encoding speed, velocity, and head direction cells,^20^ these may be sufficient to enable successful navigation. That is, although firing rates and the number of units in the fish telencephalon are low compared to the mammalian navigation system. Importantly, HP cues provide fish with a reliable estimate of absolute depth, rather than a full three-dimensional spatial representation.

Behavioral and electrophysiological evidence indicates that HP signals are primarily used for vertical positioning and depth-based homing, constraining navigation along the vertical axis without encoding horizontal location or traveled distance. As such, HP constitutes a low-dimensional ecological cue that can substantially simplify navigation demands when combined with boundary-related and kinematic information. Recent work has further strengthened the evidence for spatial representations in the fish telencephalon. Particularly, Yang et al. (2024)^21^ demonstrated a population-level code for spatial location in the zebrafish telencephalon, revealing distributed neural activity patterns that correlate with the animal’s position in the environment. These findings suggest that fish possess neural substrates capable of representing space at the population level, even in the absence of canonical mammalian place or grid-cell firing patterns. Importantly, however, population-level spatial representations do not by themselves specify the mechanisms by which fish generate goal-directed navigation behavior. How such distributed codes are transformed into action selection, obstacle avoidance, and efficient trajectory planning under ecological constraints remains an open question. Notably, spatial representations such as place or grid-like activity are often discussed in the context of memory and familiar environments, whereas goal-directed navigation can also occur in novel or partially explored settings. This distinction raises the possibility that effective navigation may rely on mechanisms that do not require fully formed spatial maps.

The mechanisms underlying the ability of fish to measure distances from environmental features with BVC and to sense HP cues are not well known. However, there are several possible mechanisms for depth perception in fish,^22^ among them using visual parallax, i.e., how objects shift on the retina as they move,^23^ and optical flow^24^ that may be used to estimate distances from walls or boundaries. Furthermore, fish have a lateral line, a sensory system that detects water movements and pressure changes.^25^ Water flow patterns change near walls, which the fish may detect to perceive the proximity of nearby surfaces without seeing them. In addition, it was also suggested that fish can use their swim bladder to sense pressure (this air-filled organ changes its size proportionally with the change in HP)^26^ and is physically connected to the inner ear of fish.^27^^,^^28^ Thus, implying auditory pathways that are involved with position encoding. Last, memory can also be used, as it was shown that fish are capable of learning their environment and make shortcuts to their target.^29^ This implies their ability to path integrate, i.e., to maintain a sense of position by integrating self-motion cues.^30^

To address this gap, we designed a neuromorphic (brain-inspired) spiking neural network (SNN), which follows the Goldfish navigation framework. SNNs are considered biologically plausible as they are based on discrete spiking events rather than on the continuous and differential signals of conventional artificial neural networks (ANNs).^31^ One theoretical framework, which commonly underlies SNNs, is the neural engineering framework (NEF).^32^ NEF was implemented as *Nengo*^33^ and is commonly used for perceptual and cognitive studies as a platform for biologically plausible models.^34^^,^^35^ Although NEF-based models implementing memory and path integration to navigate were shown to work before,^8^^,^^36^ these were inspired by mammalian spatial memory studies.

**Figure 1:**
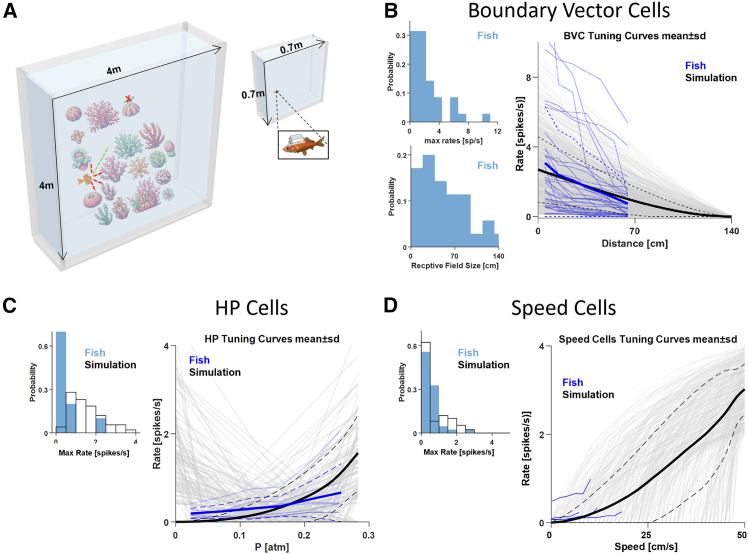
Naturally inspired modeling of the goldfish navigation system (A) Left: schematics of the virtual arena. Dashed lines are an example of BVC activity: red lines indicate the direction and distance of nearby objects, and the green line indicates a possible clear path. The Red “x” indicates the target. Right: arena schematics of a goldfish electrophysiology study used as Cohen et al.^1^ (B) BVC properties from Cohen et al. (2023)^1^ vs. their implementation in *Nengo*. Left: neuronal maximal rates (top) and receptive field sizes (bottom) of BVC. Right: neuronal (blue thin lines) vs. modeled (gray thin lines) BVC tuning curves. Thick lines and dashed lines represent mean and standard deviation, respectively. (C) Hydrostatic pressure (HP) cell properties from Cohen (2023)^17^ vs. their implementation in *Nengo*. Left: neuronal (blue) vs. modeled (black) maximal rates. Right: neuronal (blue thin lines) vs. modeled (gray thin lines) tuning curves of firing rate to HP. (D) Same conventions as in C, but for speed cells.^20^

**Figure 2:**
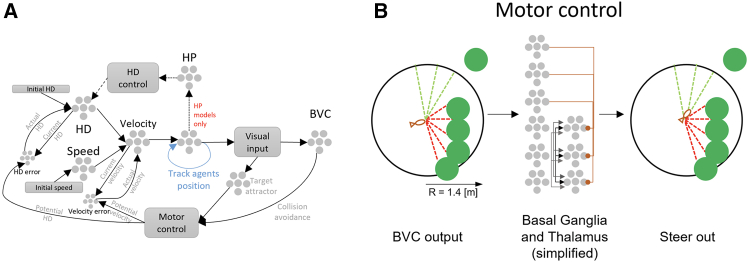
A NEF-based model of the goldfish navigation system (A) Schematics of the Nengo model used to resemble the navigation system of the fish. Cell types are in accordance with Figure 1A. Dotted clusters are neuron ensembles, and squared frames are nodes resembling external inputs to the navigation system. (B) Motor control with BVC example: If the front direction is blocked, BVC data (vector of distances and directions, dashed lines) propagates to neural models of basal ganglia and thalamus, which together output the preferred steering angle.

Here, we demonstrate fish-inspired navigation without explicit place-cell position encoding, leveraging boundary-vector and hydrostatic cues to achieve efficient spatial behavior in naturalistic arenas (Figure 1). We implemented four NEF-based navigation models inspired by the Goldfish’s natural navigation system. To computationally model the fish’s navigation system, we used experimentally derived neural tuning curves and population sizes, which were recorded from the goldfish telencephalon (Figures 1B–1D). Our model’s building blocks are BVC, HP, and kinematical cells, allowing the encoding of speed and head direction (Figure 2). We evaluated various navigation strategies, including following a random direction, a known initial azimuth, or using the target’s HP. We further evaluated our models with varying neuronal resources. Aiming to keep the modeled navigation circuit biologically plausible, we treated all of the above mechanisms as black boxes. i.e., instead of modeling these neural systems, we used the position of the virtual agent in the arena and defined accordingly, outside the simulation, the visual scene input to the BVCs system and the HP cues inputs to the HP cells. We used these cues mainly to achieve a successful navigation with no regard for the agent’s current 3D position or odometer.

## Results

**Figure 3:**
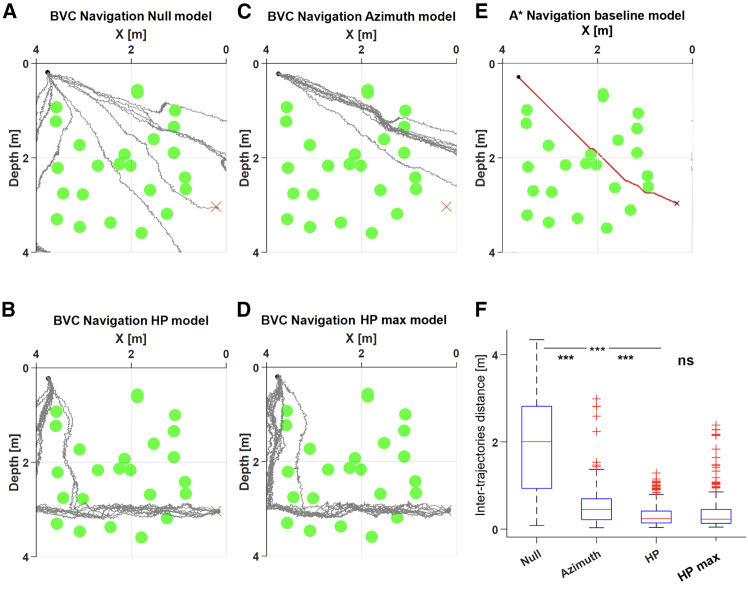
Power analysis of the different navigation strategies (A–D) Multiple trajectories (gray lines) taken by the agent to the target (red “x”) over the same environment. In each panel, a different navigation strategy was used, including the (A) Null, (B) HP, (C) Azimuth, and (D) HP_max. (E) A∗ baseline models. (F) Inter-trajectories distance (mean ± sd) among the different strategies across six different environments. ∗∗∗p < 1e−4 is the *t* test result, suggesting that the HP model performed significantly better than the others.

To test the power of different navigation strategies we simulated six environments, each of which was repeatedly tested 10 times with each of four navigation strategy models: the Null model-a reference model with no specified navigation strategy; the Azimuth model-where only the initial direction to the target is known to the agent; the HP model-where the HP in which the target is present is known to the agent; and the HP_max model, which is the same as the HP model but with 100-fold higher firing rates and a 10-fold larger neuronal population. An example environment is presented in Figures 3A–3D. After the simulations ended, we tested how similar the trajectories of each navigation strategy were. For that, we then summed the stepwise Euclidean distance (see STAR Methods) between all possible pairs (total *n* = 45 pairs) of the 10 trajectories, and repeated this over the four navigation strategies and in all six environments combined. This yielded a set of inter-trajectory distance values for each navigation strategy (Figure 3F), where low values indicate high similarity between the trajectories. We found the similarity was much higher (i.e., the distance was much lower) for the HP model compared to both the Null and Azimuth models (p < 1e−4 for both, *t* test). Further, although in the HP_max model, firing rates were much higher and many more neurons were used, no difference was shown compared to the HP model. This implies the efficiency of the relatively simple navigation system in the brain of fish.

**Figure 4:**
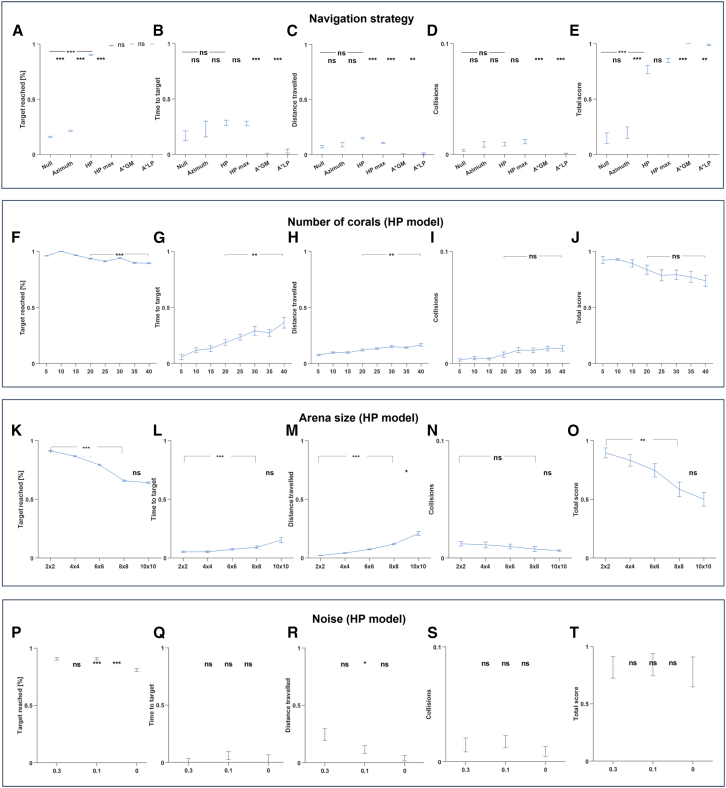
Model comparison (A–E) Navigation strategy comparison: For each of the navigation strategies (Null, Azimuth, HP and HP_max), as well as the A∗-global map (GM) and A∗-local partial (LP) planners, we specified error bars (mean ± SE) for the (A) portion of successful trials (out of total *n* = 50 simulations). For the successful trials only, presented are: (B) normalized time to target, and (C) normalized distance traveled. (D) Collisions portion of the total trajectory. (E) Total score of each navigation strategy. Failed trials were folded to score = 0. ns, *p* > 0.05; ∗,p < 1e−2; ∗∗, p < 1e−3; and ∗∗∗, p < 1e−4; *t* test *p*-values suggesting the HP performed significantly better than the two other models, and not statistically different from the HP_max model. (F–J) The HP model score does not decrease with task complexity. Same conventions were used as in A-E. As the number of corals increases from 5 to 40, as expected, the time (G) and distance (H) to target increase, as well as a slight increase in collision rate (I). Nevertheless, the total score (J) did not decrease. (K–O) HP model score scalability test. Same conventions were used as in A, B, C, D, and E. (P–T) HP model effect of noise. Same conventions were used as in A, B, C, D, and E.

We further tested the different navigation strategies for 50 different environments: each with a different starting position, target position, and coral distribution in the arena. To quantify the performance of each strategy, we calculated the score value composed of the following values (normalized, see STAR Methods): Target reached (Figure 4A), time to target (Figure 4B), distance traveled (Figure 4C), and collisions (Figure 4D), yielding the total score (Equation 1 and Figure 4E).

Although in the few successful simulations with null and azimuth navigation strategies, time (Figure 4B) and distance travelled (Figure 4C) were not statistically different when compared to the HP strategy, the total score (Figure 4E) was much higher (p < 1e−4, *t* test) when using HP as a path control parameter. The collision rate (Figure 4D) was similarly low for all strategies, consistent with the model design since all models used the BVC activity for collision avoidance. Here, again, the efficiency of the fish navigation system was exemplified, as the total score of the HP model was similarly high to the corresponding score of the HP_max model, although the number of neurons and firing rates were much lower in the HP model.

Across 50 matched environments, pairwise contrasts showed that the HP model markedly outperformed both the Null and Azimuth baselines. Success-rate differences were large (Cohen’s d = 2.03, *p* < 10^−14^ vs. Null; d = 1.75, *p* < 10^−11^ vs. Azimuth), with equally large improvements in total distance traveled (d ≈ 1.5 and 1.1, *p* < 0.02). Collision-rate reductions were moderate (d ≈ 0.6, *p* < 0.005). HP and HP_max_ achieved statistically indistinguishable performance on most metrics (|d| < 0.4, *p* > 0.3) despite HP using ∼10× fewer neurons and 1000× fewer spiking events, confirming efficiency without loss of accuracy. One-way ANOVAs (factor = strategy) yielded partial η^2^ = 0.38–0.62, indicating large effect sizes across all navigation outcomes even after Holm-Bonferroni correction (familywise α = 0.05).

To assess how our model performs relative to a traditional planner, we compared it with an A∗ algorithm (see STAR Methods). Both A∗ modes (global and local) successfully solved all 20 test environments. The global A∗ condition produced the shortest and most direct trajectories, as expected from an algorithm with full map access. The local A∗ mode, operating under the same limited sensory range as our SNN, required frequent replanning and generated slightly longer routes and slower arrival times. Numerically, A∗ with global map access achieved normalized time and distance values close to zero, indicating optimal trajectories, whereas the local-sensing A∗ exhibited small positive deviations (time to target ≈0.03; distance traveled ≈0.007) and a minor collision rate (≈0.0002).

While the A∗ algorithm achieved higher numerical efficiency, it does so by leveraging full map information and idealized search heuristics, conditions that do not exist in biological systems. Our model, by contrast, operates under limited sensory input and without explicit map representation, yet achieves reliable goal-directed navigation. This demonstrates that efficient goal-directed behavior can emerge under constrained task conditions from distributed spiking dynamics without requiring explicit map-like representations, offering a more biologically grounded mechanism for navigation than classical path planners. We further tested the HP model for its dependency on the task complexity (Figures 4F–4J) and on the arena size (Figures 4K–4O). For the task complexity case, 50 different environments were repeatedly tested, each time with a different number of corals in the range between 5 and 40 corals. As expected, the time (Figure 4G) and distance (Figure 4H) to target gradually increased with the difficulty of the task (p < 1e−3 for both cases, *t* test between results for environments with 20 and 40 corals). In contrast, the number of collisions remained low and did not differ between the two conditions (Figure 4I; *p* = 0.11), and the resulting navigation score was comparable across difficulty levels (Figure 4J; *p* = 0.15).

For the arena size case, 50 square arenas of each of the sizes 2 m × 2 m, 4 m × 4 m, 6 m × 6 m, 8 m × 8 m, and 10 m × 10 m were simulated and tested. The number of corals and simulation duration increased proportionally with the size of the arena. We found that the target was reached more in smaller arenas (Figure 4K), with a plateau around 8 m × 8 m (*p* = 0.1, *t* test between arenas of 8 m × 8 m and 10 m × 10 m in size). Consistent with the scaling of the environment, the relative searching time and distance increased with arena size (Figures 4L and 4M; p < 1e−2 and p < 1e−4, respectively; *t* test between arenas of 2 × 2 m and 8 × 8 m in size).

Collision rate stayed low regardless of arena size (Figure 4N; p < 1e−4; *t* test). In spite of the decrease in total score from 2 m × 2 m arena to 8 m × 8 m arena (p < 1e−4, *t* test; Figure 4O), scores remained above 0.55 on average across all conditions and did not further decrease when increasing arena size to 10 m × 10 m (*p* = 0.25, *t* test). This suggests the HP model has some scalability with the arena’s size.

To model sensory and state-estimation uncertainty, Gaussian noise (σ = 0, 0.1, 0.3) was injected into the positional state variable, which integrates boundary-related distance estimates and self-motion cues and serves as the effective input to the BVC population, Figures 4P–4T. Collision rate and time to target did not differ significantly across noise conditions (all comparisons, ns). In contrast, the success rate was significantly lower in the no-noise condition (σ = 0) compared to both noisy conditions (σ = 0.1, 0.3; *p* < 0.001). Distance traveled showed a modest effect of noise, with significantly shorter paths at σ = 0.1 compared to σ = 0.3 (*p* < 0.05), while other pairwise comparisons were not significant. The composite performance score did not differ significantly across noise levels. Together, these results indicate that moderate levels of noise improve the reliability of goal acquisition without substantially affecting collision frequency or navigation time, while higher noise levels primarily impact path length rather than overall task success.

**Figure 5:**
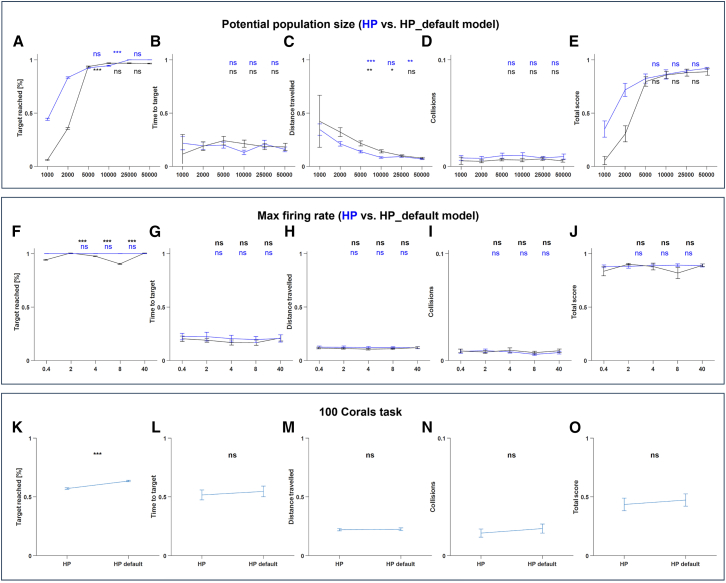
HP vs. HP_default model We compared the HP model with the HP_default model performances to check how the tuning to natural neuronal characteristics (HP model) affected the results compared to the random tuning used in *Nengo*. (A–E) Population size comparison. Same conventions were used as in Figures 4A–4E. Similar plateaus were observed for the different models’ total score as the potential population size increased from 1,000 to 50,000 neurons for the HP (blue curves) and HP_default (black curves) models. (F–J) Maximal firing rate comparison. Same conventions were used as in A, B, C, D, and E. Stable high scores were observed for the different models’ total score as the maximal firing rates were increased from 0.4 to 40 spikes/s for the HP (blue curves) and HP_default (black curves) models. (K–O) High complexity task. Same conventions were used as in A, B, C, D, and E. Both models performed similarly in a high complexity task, including 100 corals in the arena.

We further tested the importance of neuronal tuning in the natural characteristics of neurons as observed in fish. These included the distribution of maximal firing rates for BVC and HP units, as well as the distribution of receptive field sizes for the BVC. Hence, we compared the HP model with the HP_default model, where no manual tuning was done, but the random tuning as defined by *Nengo* (see STAR Methods). We first varied the total number of neurons available for the two models in the range [1000, 50,000]. Each population size was tested in 30 different environments (Figures 5A–5E). In both cases, plateaus of similar success rates (Figure 5A) and total score (Figure 5E) were observed in around 5000 neurons. Nevertheless, the main difference between the models was observed in the small population size cases: In both 1000 and 2000 neurons, success rates (and corresponding total scores) were much higher for the tuned (HP) model (p < 1e−4, *t* test). We then repeated the comparison between the HP and HP_default models, but with the same number of neurons (*n* = 10,000) and with varying maximal firing rates for all neurons in the range 0.4–40 spikes/s (Figures 5F–5J), each of which was tested in 30 different environments. In both cases, a high success rate was observed across all firing rates, yet with a more stable success rate and total score curves for the tuned (HP) model (Figures 5F and 5J, blue curves). These results again manifest the high and stable natural navigation capabilities even in small systems, as in the fish brain.

Finally, we evaluated the two models in 50 high-complexity arenas (100 corals each) to test whether the tuning of BVCs to their natural characteristics would change their ability to avoid collisions (Figures 5K–5O). As in the population size test (Figure 5D) and in the maximal firing rate test (Figure 5I), a similarly low collision rate was observed for the two models. This may suggest that the natural tuning of BVCs is important for their ability to encode or memorize position rather than for collision avoidance.

## Discussion

Various computational models were designed to follow neural navigation systems. These models are often governed by two main building blocks as the neural substrate of navigation: the place cell as position control, and the grid cell as odometer.^8^^,^^9^ Together, these cell types were suggested to be key players underlying the mental map of space in the mammalian brain, termed a cognitive map.^37^ This map is described as a mental GPS, enabling successful navigation while even supporting shortcuts.^29^

Nevertheless, the typical place and grid cells recorded in small, controlled, laboratory settings failed to scale up to natural environments, as their behavioral patterns were found to differ.^11^^,^^12^^,^^13^ In fish, the largest vertebrate class, early studies did not report neurons exhibiting the stereotypical, periodic grid-like or localized place-field patterns described in mammals. Instead, spatial encoding was suggested to rely primarily on boundary-related signals and HP cues, consistent with BVC-like responses and depth-related representations.^1^^,^^17^ More recent work has reported population-level spatial representations in fish, including place- and grid-like correlates under specific experimental conditions.^21^ However, these representations appear less canonical and more context-dependent than their mammalian counterparts, leaving open the question of their functional role in navigation.

Against this background, the central result of the present work is that a biologically grounded navigation system based on BVCs, HP cues, and simple kinematic signals is sufficient to support robust goal-directed behavior in complex, cluttered environments. Across a wide range of arenas, obstacle densities, and spatial scales, the model reliably reached the target without relying on explicit place-cell or grid-cell representations or maintaining a global spatial map. Importantly, successful navigation did not depend on increased neuronal resources or elevated firing rates, indicating that efficient behavior can emerge from sparse, low-rate population dynamics consistent with those observed in the fish telencephalon. Notably, introducing moderate levels of noise did not degrade performance and, in fact, improved the reliability of goal acquisition relative to the fully deterministic regime, highlighting the robustness of the proposed control architecture under biologically realistic variability.

The improvement in goal-reaching reliability under moderate noise suggests that fully deterministic navigation dynamics may be susceptible to trapping behaviors in complex environments, such as limit cycles or dead-zone effects induced by local symmetries. Introducing stochastic perturbations to the positional state can reduce sensitivity to such dynamical artifacts, facilitating escape from suboptimal regimes without requiring additional computational resources. Importantly, this effect was observed without increases in collision rates or navigation time, indicating that moderate variability enhances robustness rather than degrading navigational efficiency.

These results were obtained using a neuromorphic SNN grounded in electrophysiological data from the goldfish telencephalon and implemented under biologically realistic constraints. The neural design was tuned based on *in vivo* recordings from the goldfish telencephalon. Whereas most artificial models of the mammalian navigation system are composed of place and grid cells, with neuronal populations numbering in the thousands and exhibiting high firing rates (dozens of spikes/s), navigation in fish relies on markedly lower firing rates and smaller neuronal populations.^1^^,^^20^ Accordingly, we modeled a biologically plausible SNN composed of BVCs, HP cells, and kinematic representations (speed, head direction, and velocity), using population sizes, firing rates, and tuning curves observed in the goldfish. To address scalability, we simulated a ×32 digital twin relative to the arena used in the electrophysiological study. Whereas most artificial models of the mammalian navigation system are composed of place and grid cells, with neuronal populations composed of thousands of cells, exhibiting high firing rates (dozens of spikes/s), in fish, the case is different: Firing rates as well as neuronal population sizes are distinctively lower.^1^^,^^20^ Therefore, to allow for a biologically plausible neural design, we used the population sizes, firing rates, and tuning curves observed in the goldfish to model an SNN, composed of BVC, HP cells, and kinematical cells (speed, HD, and velocity). To address the scalability issue, we simulated an x32 digital twin in comparison to the electrophysiology study’s arena.

The virtual fish faced a navigation task: finding a food pellet in an arena covered with virtual corals. We compared several navigation strategies to test whether they can underlie the successful navigation abilities observed in fish.^15^^,^^18^ While using HP as a single cue, it resulted in a slightly longer path from the initial agent’s position to the target. This strategy was found to be better than the null (random direction) and azimuth (knowing the initial direction to the target) strategies. We found that navigation using HP cues only can be used to repeatedly solve underwater mazes with similar trajectories (Figure 3). The high success rates observed did not increase with higher firing rates and large population size (Figures 4A–4E). Further, the high success rates of the HP navigation model showed a stable response with respect to task complexity (Figures 4F–4J), and a scalable response for arenas in different sizes (Figures 4K–4O). We further compared our biologically plausible model (which was tuned in accordance with the goldfish telencephalon recordings) to a non-tuned random model. We found that the tuned model performed better in small populations (Figures 5A–5E) and was more stable with various firing rates (Figures 5F–5J). Interestingly, simulating 45 s of virtual navigation required approximately 200 s of wall-clock time on the GPU. Although not real-time, the fish-tuned model operates at low firing rates (<4 spikes/s) with about 10,000 neurons, whereas the HP_max_ control uses roughly 10× more neurons and allows firing rates up to 400 spikes/s. Consequently, the expected number of spiking events, used here as a neuromorphic energy proxy, is about 1000× lower in the fish-tuned model, consistent with comparable performance at substantially reduced event load.

Although we have shown that robust goal-directed navigation can be achieved without explicit place- or grid-cell representations, an important question concerns how these findings relate to recent physiological evidence for spatial coding in the fish brain. Recent work has reported population-level spatial representations in the zebrafish telencephalon, demonstrating that distributed neural activity can encode information related to the animal’s location, even in the absence of canonical, stereotyped place- or grid-cell firing patterns.^21^ However, the presence of spatially informative population codes does not, by itself, imply the existence of an explicit navigational map or specify how spatial information is translated into action. Such population-level representations may support multiple functions, including state estimation, contextual modulation, or behavioral biasing, without necessarily serving as a metric substrate for navigation.

From this perspective, our model explores how relatively low-dimensional ecological cues, such as boundary-related signals and HP, can support action selection without requiring an explicit spatial map. One plausible interpretation is that BVCs provide a low-level geometric basis from which higher-order, population-level spatial representations, such as those reported by Yang et al. (2024),^21^ may emerge through integration with self-motion cues. Under this view, place-like or grid-like responses observed in fish would reflect downstream transformations of boundary-based and self-motion information rather than primary substrates for navigation. This interpretation is consistent with the prominence of boundary-related coding in fish and with the context-dependent, non-canonical nature of reported grid- and place-like correlates.

Together, these results demonstrate that ecologically grounded cues such as boundary-related signals and HP may be sufficient to support reliable navigation behavior in depth-constrained tasks, without requiring explicit map-like representations within the model. At the same time, this approach relies on abstracted sensory signals and simplifying assumptions that shape the interpretation of the model’s scope.

Visual and hydrostatic-pressure inputs are modeled at an abstract level, without explicitly representing intermediate sensory processing (e.g., optic tectum pathways or swim-bladder/inner-ear transduction). Despite this simplification, the model captures key aspects of behavioral-level navigation dynamics. Although the agent receives an initial coarse directional bias toward the target, this signal is not updated after obstacle avoidance or depth changes. All trajectory corrections in the horizontal plane emerge from local boundary-based interactions rather than from global position estimation or azimuth recalculation. In natural settings, HP cues are unlikely to be sufficient for navigation on their own, as they primarily constrain movement along the vertical axis. In our model, HP is therefore not treated as a general spatial signal, but as an ecological prior that reduces the dimensionality of the search space and biases action selection toward task-relevant depth levels. This abstraction allows us to isolate the computational role of HP cues while remaining consistent with their known biological limitations. Furthermore, clearly, in nature, fish cannot rely merely on HP to get to their home range or feeding ground. One option is that fish use BVC not only for collision avoidance but also to calculate position gradually by encoding distances from multiple salient features in the environment and using these features as key milestones in their navigation task. Although that is a valid option, further research is needed to model and test this navigation strategy (as well as its combination with the HP strategy) to better model the fish navigation system.

To conclude, we designed an SNN as a navigation framework, independent of local encoding of position with a relatively low neuronal population size and firing rates, as observed in real electrophysiological studies. Our results suggest that smaller navigation system models, as in fish, may still be energy-efficient and a valid alternative to the typical mammalian ones. Our model, aimed at solving underwater mazes, is not aimed at depreciating alternative models. Rather, it gives an important aspect for the comparative approach between model animals, as well as for the evolutionary understanding of spatial memories in general.

### Limitations of the study

This study adopts several simplifying assumptions that constrain the interpretation and generalizability of the results. First, sensory processing pathways were modeled at an abstract level, with visual boundary signals and HP cues provided as pre-computed inputs rather than emerging from detailed sensory transduction models. As a result, the present model does not address how boundary-related or pressure-related neural responses are generated in biological systems.

Second, navigation was evaluated in environments in which the agent received a fixed initial directional bias toward the target. This bias reduces the dimensionality of the search problem and should not be interpreted as evidence that HP cues alone are sufficient for unrestricted navigation in natural environments.

Third, although the model does not provide the controller with explicit access to Cartesian position variables, positional state variables were used internally to generate sensory inputs. This reflects a standard simulation approach but introduces a level of abstraction that may differ from biological implementations.

Finally, the present work focuses on demonstrating computational sufficiency rather than biological exclusivity. Fish navigation likely relies on multiple interacting sensory modalities, including vision, optic flow, lateral line sensing, and memory-based strategies, which were not modeled here.

Future work may extend the framework to incorporate adaptive directional biases, learning mechanisms, and richer sensory processing models.

## Resource availability

### Lead contact

Further information and requests for resources should be directed to and will be fulfilled by the Lead Contact, Elishai Ezra Tsur (elishai@nbel-lab.com).

### Materials availability

This study did not generate new biological materials.

### Data and code availability

All simulation scripts and model implementations are available in the repository: https://osf.io/ypmfq/overview (DOI: https://doi.org/10.17605/OSF.IO/YPMFQ).

Any additional information required to reproduce this work is available from the lead contact upon request.

## Acknowledgments

This work was supported by the 10.13039/501100024250Israel Innovation Authority as part of the NeMo Consortium. The authors would like to thank the members of the NeuroBiomorphic Engineering Lab (NBEL) for the insightful discussions.

## Author contributions

Conceptualization: all authors contributed; methodology: L.C.; software: L.C.; validation: L.C and H.C.D; formal analysis: L.C.; investigation: L.C. and H.C.D.; writing – original draft: all authors contributed; writing – review and editing: H.C.D. and E.E.T.; visualization: L.C; supervision: E.E.T.; project administration: E.E.T.; funding acquisition: E.E.T.

## Declaration of interests

The authors have declared that no competing interests exist.

## STAR★Methods

### Key resources table

**Table:** 

REAGENT or RESOURCE	SOURCE	IDENTIFIER
**Software and algorithms**		

Nengo 4.0.0	Bekolay et al., 2014	https://www.nengo.ai
Python 3.9.0	Python Software Foundation	https://www.python.org/
MATLAB	MathWorks	https://www.mathworks.com/
A∗ (A-star) algorithm	Hart et al., 1968	doi: https://doi.org/10.1109/TSSC.1968.300136

**Other**		

NVIDIA A100 GPU (80GB VRAM)	NVIDIA	https://www.nvidia.com/en-us/data-center/a100
Goldfish navigation codebase	This paper	https://osf.io/ypmfq/overview (DOI: https://doi.org/10.17605/OSF.IO/YPMFQ)

### Method details

#### Biological inspiration for neural representations

Neural representations used in this model were derived from previously published electrophysiological studies of the goldfish telencephalon, a brain region considered homologous to the mammalian hippocampal formation.^1^^,^^17^^,^^20^

In these studies, extracellular neural activity was recorded using wireless implants while fish freely explored shallow and vertical quasi-two-dimensional environments. The experiments identified spatially modulated neural populations including boundary vector cells, hydrostatic pressure cells, head direction cells, speed cells, and velocity cells.

Boundary Vector Cells encode distance and direction relative to environmental boundaries, while hydrostatic pressure cells encode vertical spatial position. These experimentally measured tuning properties informed the distributions of receptive field parameters and firing rates used in the computational model.

No new biological experiments were performed in the current study.

#### Virtual navigation task design

A scaled-up digital twin (x32) of the experimental setup was designed, creating a virtual arena of 4 m (depth) x 4 m (length). The environment included a stochastically placed target and starting position in opposite quadrants, with 20 to 30 circular obstacles (corals) of 0.15 m radius distributed randomly to simulate a natural underwater maze. The agent aimed to reach the target as quickly as possible without collisions, with a maximal speed of 0.5 m/s.

#### Neural populations

The navigation model includes neural populations inspired by electrophysiological recordings in the goldfish telencephalon.^1^^,^^17^^,^^20^ These include Boundary Vector Cells (BVC) and Hydrostatic Pressure (HP) cells forming the spatial encoding component of the navigation system, as well as Speed cells, Head Direction (HD) cells, and Velocity cells forming the kinematic component.

Population sizes were determined according to experimentally observed proportions of spatially modulated neurons recorded in the goldfish telencephalon.^1^^,^^17^^,^^20^ The total number of neurons in the model was set to approximately n = 10,000 neurons, consistent with estimates of the number of neurons involved in spatial navigation in the fish pallium.

For example, 35 out of 196 recorded neurons (17.85%) were classified as Boundary Vector Cells, yielding a modeled population of 1785 BVC neurons. Other neural populations (Speed, Head Direction, and Velocity cells) consisted of approximately 1500 neurons each.

#### Boundary vector cell tuning

Boundary Vector Cells encode egocentric distance and direction relative to environmental boundaries. To reproduce experimentally observed tuning properties, we used electrophysiologically measured firing rate curves of BVC responses to distance from environmental boundaries in the goldfish telencephalon^1^ (Figure 1B).

We fitted skew-normal distributions to both peak firing rates (Figure 1B, top left) and receptive field sizes (Figure 1B, bottom left). These distributions were then used to generate modelled BVC tuning curves with corresponding intercept and amplitude parameters (Figure 1B, right). Mean and standard deviation of the fitted distributions matched experimentally observed variability across recorded neurons.

Directional sensitivity was implemented using an ensemble-array representation in which each ensemble encodes boundary distance along a preferred direction. Directional tuning covered the full angular space using a uniform resolution of: Δθ = 15° resulting in 24 directional bins.

The same procedure was applied to model Hydrostatic Pressure cells, using experimentally measured tuning curves corresponding to vertical spatial position^1^ (Figure 1C).

#### Neuron model parameters

Boundary Vector Cells were implemented using the spiking Rectified Linear neuron model provided in Nengo. This neuron model produces approximately linear tuning curves across the receptive field, consistent with experimentally observed BVC response profiles, and better matches measured tuning functions than standard leaky integrate-and-fire (LIF) neurons.

Speed cells encode absolute swimming speed of the agent, Head Direction cells encode allocentric orientation, and Velocity cells encode the velocity vector obtained by combining speed and direction signals. These neural populations were implemented using Nengo’s leaky integrate-and-fire neuron model.

Neuron gain parameters were selected to match experimentally observed firing rate ranges in the goldfish telencephalon:

0.1–4 spikes/s.^1^^,^^20^

Hydrostatic Pressure cells were implemented using the same neuron model and tuning parameterization approach as Boundary Vector Cells, adjusted to match experimentally observed HP tuning curves.^17^

#### Navigation strategies

To evaluate the contribution of biologically motivated spatial signals to navigation performance, we implemented several navigation strategies that differed in the information available to the agent regarding target location.

#### Null model

As a baseline condition, we implemented a null model in which the agent had no information regarding the target location. The initial heading direction was sampled randomly within ±45° of the true target direction. The agent updated its swimming direction only when obstacles were detected in front of it. Collision avoidance and speed control were implemented using Boundary Vector Cell (BVC) activity, as described above, and were identical across all navigation strategies.

#### Azimuth model

In the azimuth model, the agent was provided with the initial allocentric direction to the target location. Apart from this initial directional information, all navigation dynamics were identical to the null model. In particular, obstacle avoidance and speed modulation were governed solely by BVC activity.

#### Hydrostatic pressure model

In the hydrostatic pressure (HP) model, the agent was additionally provided with vertical spatial information corresponding to the hydrostatic pressure of the target location. The agent could therefore estimate the relative vertical position of the goal using HP-sensitive neural populations.

At the beginning of each trial, the agent was assigned a coarse binary directional bias indicating whether the target was located to the left or right relative to the initial starting position. This directional bias was represented as a discrete angular prior:

$$\theta_{HP}\in \left\{0,\pi \right\}$$

The bias remained fixed throughout the trial and did not depend on the agent’s current position, accumulated displacement, or interactions with obstacles. The directional bias was provided only at initialization and remained fixed throughout the trial. The controller did not update azimuth estimates based on accumulated positional information, path integration, or environmental interactions. As a result, horizontal navigation adjustments emerged from local boundary-based interactions rather than from continuous global heading correction. This constraint reduces the dimensionality of the search problem and reflects the specific task conditions evaluated in this study rather than unrestricted spatial navigation.

#### HP-max model

To evaluate the influence of neuron model assumptions on navigation performance, we implemented an additional HP-based model using standard leaky integrate-and-fire (LIF) neurons with higher firing rates and increased population size.

In this variant:

maximum firing rate = 400 spikes/s

number of neurons = 10 × baseline population size

All neural populations were implemented using standard LIF neuron models with default parameter settings in Nengo.

This model allowed us to evaluate whether navigation performance depended on biologically realistic firing rate regimes or could be reproduced using conventional high-rate spiking neuron models.

#### HP-default model

To evaluate the importance of experimentally constrained tuning parameters, we implemented a variant of the HP model in which Boundary Vector Cell receptive field parameters were not constrained by experimentally fitted skew-normal distributions.

Instead, intercepts determining receptive field size were sampled from Nengo’s default normally distributed intercept distribution. Similarly, maximal firing rates for both BVC and HP neural populations were sampled from the default Nengo parameter distributions.

All other model components remained identical to the biologically constrained HP model.

This comparison allowed us to isolate the contribution of experimentally observed tuning distributions to navigation performance.

#### NEF-based navigation architecture

The navigation model was implemented using the Neural Engineering Framework (NEF; Eliasmith & Anderson, 2003) in the Nengo neural simulator (Nengo 4.0.0). NEF provides a principled framework for constructing spiking neural networks that approximate dynamical systems using neural population codes and synaptic weight optimization.

Neural populations encoded low-dimensional task variables relevant for navigation, including boundary-related signals, hydrostatic pressure (HP), head direction, speed, velocity, and spatial position. Synaptic connection weights were computed using standard NEF decoding methods to approximate nonlinear transformations between represented variables.

All neural populations consisted of leaky integrate-and-fire (LIF) neurons unless otherwise specified. Synaptic connection weights were fixed prior to simulation.

Two dynamical systems were implemented using NEF principles: neural integrator and neural attractor.

The neural integrator was implemented using recurrent connectivity approximating temporal accumulation of velocity signals into spatial position.

The neural attractor was implemented using recurrent dynamics producing stable goal-directed trajectories.

The overall architecture is illustrated in Figure 2.

#### Kinematic representation

The virtual navigation task was initialized as the agent is placed in a square arena of size 4×4 m, and it starts to move at a maximal speed of *S*_0_(*t*)=*v*_*max*_= 0.5 [*m*/*s*], which was encoded by the ensemble of speed cells. The initial HD was set according to the navigation strategy as described above and was encoded by the ensemble of HD cells. The agent’s dynamics are governed by translational velocity $\vec{v}\left(t\right)=\left(v_{x}\left(t\right),v_{y}\left(t\right)\right)$ , which is derived from the speed component *s*(*t*) and head direction *θ*(*t*):

$$\vec{v}\left(t\right)=s\left(t\right)\cdot \left[\cos\;\left(\theta \left(t\right)\right),\sin\;\left(\theta \left(t\right)\right)\;\right]$$

where the translational velocity was encoded by a third ensemble- the velocity cells.

To resemble natural brain processes, the connection between neuronal ensembles in Nengo enables a synaptic time constant as a low-pass filter over time of the synaptic current. Here, we used a synaptic time constant of *τ*_1_=20 [*ms*] for the conjugation of the speed and HD cells into the velocity cells. The output of the velocity cells was then propagated to a fourth ensemble, which calculated the vector-step-size per simulation step. The step-sizes were then integrated (synaptic time constant of *τ*_2_=100 [*ms*]) to calculate the current position of the agent, $\vec{p}\left(t\right)$, according to the differential equation $\frac{d\vec{p}\left(t\right)}{dt}=\vec{v}\left(t\right)$. In the NEF framework, this integration is implemented using a recurrent neural connection with identity feedback and an input scaled by the synaptic time constant *τ*_2_ to change units from [m/s] to [m]. This ensemble, although encoding position, was not set as typical mammalian place-cells, which are characterized by a Gaussian tuning to space with high firing rates and a large number of neurons in the population. Rather, it was characterized by Nengo's default (LIF) neuron type, with the same size as the previous ensembles (1500 neurons) and low firing rates (up to 4 [spikes/s]). Further, unlike place-cells, which are used for navigation, we only used this data to define the visual scene (externally of the neural simulation) and the input to the HP cells in the relevant models, as described below.

Importantly, the positional state variable computed by the neural integrator was not directly available to the navigation controller as an explicit spatial representation. Instead, this variable was used solely to generate sensory inputs provided to the model, specifically boundary-related distance signals and hydrostatic pressure cues. The controller itself therefore operated exclusively on boundary-derived and hydrostatic inputs, without direct access to Cartesian coordinates, accumulated displacement, or an explicit global spatial map. This distinction ensures that navigation performance does not rely on internal availability of ground-truth position variables.

#### Hydrostatic pressure-based control

In HP and HP-max models, the vertical component of position was projected to Hydrostatic Pressure neural populations using synaptic time constant *τ*_1_.

HP activity modulated head direction via a control node determining whether the target was located above or below the current position.

When the difference between agent depth (*P*_*z*_)and target depth (*G*_*z*_)was below:

|*P*_*z*_−*G*_*z*_| < 0.2 m

the head direction bias was set to: *θ*_*HP*_ ∈ {0, π/2}

depending on the relative position of the goal.

#### Visual input and attractor dynamics

In all models, the visual scene node was defined in accordance with the positional signal above. This was done externally from the simulation, under the model assumption that vision is a black box. Although several studies modeled how fish process visual information in the brain,^38^^,^^39^^,^^40^^,^^41^ we decided to keep the visual scene as given input to our model for simplicity.

The visual scene node in our model included the distances from all the corals that ranged up to 1.4 m from the agent's current position and the distance from the target. Therefore, this signal was then propagated in two pathways: as an input to the BVC system and as an input to the target attractor- a binary variable that states whether the target is visual or not. In the latter case, when reaching the task's target area, a motor command was sent to create a fast and direct movement of the agent towards the target. The computational model for the attractor was defined as:

$$\frac{d\vec{p}\left(t\right)}{dt}=\vec{v}\left(t\right)+\vec{G}\left(t\right)$$

$$\vec{G}\left(t\right)=\{\vec{g}-\vec{p}\left(t\right),if\;\left\|\vec{g}-\vec{p}\left(t\right)\right\|<r_{vis};\;0,otherwise$$

Note that the attractor is active only when the food is visible, i.e., when the goal location $\vec{g}$ was within a predefined visibility radius *r*_*vis*_=0.3 [*m*].

#### Boundary vector cell encoding

To model the BVC system, we modeled egocentric boundary detection using BVCs with angular resolution Δ*θ*=15°, yielding *N*_*BVC*_=24angular bins. To implement them in Nengo, BVCs were defined as an ensemble-array, i.e., an array of identical neuronal ensembles, each of which encodes a specific angle and distance to the nearest obstacle if it exists in the range of the BVC's maximal receptive field of 1.4 [m]. To compute the egocentric visual input that drives Boundary Vector Cell (BVC) activity, we first determine which angular area of the visual field is influenced by each nearby coral. Each coral is treated as a circular object with radius *r*_*coral*_, and its angular extent in the agent’s visual field is estimated by its aperture, given by $2\cdot \arctan\left(\frac{r_{coral}}{d}\right)$, where *d* is the Euclidean distance from the agent to the coral center. This aperture determines the number of angular bins *N*_*i*_ on which the coral projects in the BVC array. For each of these affected bins, we then compute the precise distance *d*_*i*_(*t*) to the coral along that direction. The sensory input (visual input) to BVCs is encoded as:

$${BVC}_{i}\left(t\right)=f\left(d_{i}\left(t\right)\right),i=1,\ldots ,N_{BVC}$$

where *d*_*i*_(*t*) is the distance in direction *θ*_*i*_ and *f* is the tuning curve.

Both BVC and the attractors output propagated through the motor-control system (described below). The motor control output defines a potential velocity to the agent, which continues to navigate in this velocity while searching for the target. The simulation ended when the agent reached the target. In cases where the agent could not find the target, the simulation was set to end after 45 seconds.

#### Motor control system

Encoding distance and direction to avoid collisions was implemented in previous studies וusing Laser readings (LiDAR^42^). This method uses the LiDAR readout, i.e., paired vectors of distances and angles from the object to all nearby visual stimuli (such as obstacles), as an input to a decision-making process that includes speed control and steering if needed. The clearest path is chosen using a “winner-take-all” mechanism^39^ implemented by Nengo's Thalamus and Basal ganglia (BG) model. Although different for fish compared to mammals, still the BG and thalamus-like structures in fish play an important role in motor control and decision making, and projecting their outputs to the pallium,^43^^,^^44^^,^^45^ which is suggested as hippocampal homologue in its importance for navigation.^46^^,^^47^ We adapted this model here and used the BVC activity as input to the BG-Thalamus circuit, followed by a temporal low-pass filtering to reduce noise (Figure 2B). This enabled slowing down ahead of corals, choosing the clearest path nearby, and accelerating to maximal speed, when possible, throughout the navigation task.

To derive the potential velocity in each spatial axis, we calculated the relative distance $\hat{d}=\frac{d-\epsilon }{D-\epsilon }$ where d is the distance from a coral found in front of the agent (i.e., *d*_0_(*t*) above), *ϵ*=0.03 [*m*] which denotes the minimum safety distance and *D*=0.4 [*m*] critical distance to start slowing down in front of obstacles. The agent’s speed along each axis *a*∈ {*x*,*z*} was then calculated as:

$$v_{a}^{new}=\left\{ \begin{array}{c} \min(\;\left(|v_{a}|+v_{\epsilon }\right)\;\cdot \;\hat{d},|v_{a}|)\;if\;d\;<\;D\\ \min\left(v_{a}+dc\cdot \tau_{2},v_{\max}\right)\;otherwise\; \end{array}\right.\;\text{;}$$

where $dc=3\frac{m}{s^{2}}$ is the deceleration coefficient.

The direction component and speed folding to the range [0,*v*_*max*_] were integrated with *v*_*a*_ to derive a potential velocity:

$$v_{a}^{potential}=sign\left(v_{a}\right)\cdot \min\left(\max\left(\left|v_{a}^{new}\right|,0\right),v_{\max}\right)$$

In cases the path in front of the agent was blocked (i.e., *d*_0_(*t*)<*D*), while slowing down to $v_{a}^{new}$ , the BVCs output was sent to the BG-Thalamus model, which yielded a steer-out angle, i.e., the clockwise angle (in the range $\theta \in \left[-\frac{\pi }{2},\frac{\pi }{2}\right]$) the agent needs to turn to get to a free pathway. Then, the output steering angle was defined as: *θ*_*steer*_=*α*·*θ*_*new*_+(1-*α*)*θ*_*old*_ where *α*=0.9 integrates 10% of the previous steering angle *θ*_*old*_, with the current steering angle *θ*_*new*_. The potential velocity, *v*_*potential*_, was then updated by rotating $v_{a}^{potential}$ according to the computed steering angle:

$${\vec{v}}_{potential}=R\left(\theta_{steer}\left(t\right)\right)\cdot v_{allocentric}\left(t\right)$$

where *R*(*θ*) is the two-dimensional rotation matrix:

$$R\left(\theta \right)=\left[\begin{array}{cc} \cos\left(\theta \right) & -\sin\left(\theta \right)\\ \sin\left(\theta \right) & \cos\left(\theta \right) \end{array}\right]$$

The potential velocity was sent to a corresponding ensemble, calculating the error between the current and the potential velocity, defining the agent's velocity as the potential one by subtracting the error from the current value.

#### Error-based trajectory update

To ensure smooth trajectory updates, both the velocity and head direction signals were corrected using low-pass filtered error terms. Specifically, the velocity error ${\vec{v}}_{error}$ and head direction error *HD*_*error*_ were computed as the difference between the potential target and the current state:

$${\vec{v}}_{error}={\vec{v}}_{potential}\left(t\right)-{\vec{v}}_{current}\left(t\right){HD}_{error}={HD}_{potential}\left(t\right)-HD_{current}\left(t\right)$$

The actual motor output was then updated via first-order differential equations:

$$\frac{{d\vec{v}}_{actual}\left(t\right)}{dt}=\frac{1}{\tau_{2}}(-{\vec{v}}_{actual}\left(t\right))\frac{{dHD}_{actual}\left(t\right)}{dt}=\frac{1}{\tau_{2}}(\left(t\right)-HD_{actual}\left(t\right))$$

#### A∗ comparison baseline

To compare our biologically inspired navigation model with a classical computational approach, we implemented the A∗ (A-star^48^) algorithm, a standard deterministic planner widely used in robotics and cognitive navigation studies. The arena was defined as 4 × 4 m (coordinates [−2, 2] × [−2, 2]) with a fixed start location at (−1.8, −1.8) and a goal at (1.8, 1.8). Each environment contained 20 circular obstacles (15 cm in radius) randomly positioned within the arena.

The A∗ planner was configured with two operating modes:

(i) Global map, in which the full obstacle map was available during planning (representing an upper bound on path optimality); and
(ii) Local partial, in which the agent could sense obstacles only within a 1.4 m radius, equivalent to the sensory field used in our SNN model, and replanned its path online at 20 Hz.

By defining A∗ in this way, we were able to directly compare our biologically inspired network with a well-established classical navigation model under equivalent sensory constraints.

### Quantification and statistical analysis

#### Power analysis

To test the power of each navigation strategy, we tested the ability to repeatedly solve the same environment. For that, five different environments were tested 10 times each with each navigation strategy (Figure 3). To quantify how each strategy performed, for each task and strategy, we stretched the 10 trajectories into vectors of the same length and calculated the Euclidian distances for each pair of trajectories using MATLAB's dynamic time warping (dtw) function. Therefore, the smaller this inter-trajectory distance is, the more similar the trajectories are. Then, the distribution of all inter-trajectory distances from the 10 simulation repetitions was calculated and compared among the navigation strategies using a t-test.

#### Target reached and success rate

A successful trial was considered a trial in which the target was reached (noted as a binary 0 or 1), regardless of the trajectory. The success rate, therefore, was the total number of successful trials out of the total trials per navigation strategy. Thus, this was a value in the range [0,1]. Error bars for this value were gained by bootstrapping the success rates per category 100 times and calculating the mean and standard error of the bootstrap set.

#### Time to target

An estimate in the range [0, 1], where 0 is the minimal possible time to reach the target (i.e., the distance between the target and the initial position of the agent divided by the maximal possible swimming speed), and 1 is the maximal possible time (i.e., the maximal simulation time of 45 s). Simulations in which the target was not reached were ignored.

#### Distance traveled

The total distance in [m] traveled from the initial agent’s position to the target was normalized such that 0 is the shortest distance possible (i.e., the Euclidian distance between the target and the initial position), and 1 is the maximal possible distance (i.e., the maximal simulation time of 45 s multiplied by the maximal speed of 0.5 m/s). Simulations in which the target was not reached were ignored.

#### Collision rate

The total collision rate was defined as follows: we first calculated the total number of samples in which the distance from the agent's position to any of the coral's central coordinates was shorter than their radius of 15 cm. Then, this number was divided by the total number of samples in the simulation. Hence, the collision rate was also in the range [0,1].

#### Total score

The total score per simulation, indicating its goodness, was in the range [0,1], and was defined as the following combination of the above four values:

$$Total\;score=Target\;reached\cdot \frac{\left(1-time\;to\;target\right)+\left(1-distance\;traveled\right)+\left(1-collisions\right)}{3}$$

#### Statistical analysis

Effect sizes were quantified as Cohen’s d for pairwise contrasts between HP and each comparison model (Null, Azimuth, HP_max_), computed from paired data across identical environment indices with pooled within-group variance. Values of d ≈ 0.2, 0.5, and 0.8 were interpreted as small, medium, and large, respectively. For multi-level factors, partial η^2^ was derived from one-way ANOVA of each metric across the four strategies. Ninety-five percent confidence intervals were obtained via bootstrap resampling (10,000 iterations). p-values were adjusted for multiple testing using the Holm–Bonferroni procedure.
